# A Strong Impact of Soil Tetracycline on Physiology and Biochemistry of Pea Seedlings

**DOI:** 10.1155/2019/3164706

**Published:** 2019-01-10

**Authors:** Małgorzata Margas, Agnieszka I. Piotrowicz-Cieślak, Dariusz J. Michalczyk, Katarzyna Głowacka

**Affiliations:** Department of Plant Physiology, Genetics and Biotechnology, Faculty of Biology and Biotechnology, University of Warmia and Mazury in Olsztyn, Oczapowskiego 1A, 10-718 Olsztyn, Poland

## Abstract

Antibiotics are a new type of contaminants found in the environment. They are increasingly used in farm animal production systems and may accumulate in crops, limiting the plant growth rate and nutritive value. The aim of this study was to determine the effects of tetracycline (TC) on physiological and biochemical properties of pea seedlings. The presence of TC in the soil during 24 hours did not result in any distinct changes of the seedlings. However, after five days (120 h) of soil TC action, the seedling appearance and metabolic activities were significantly affected. Leaves lost their green coloration as a result of a 38% degradation of their chlorophyll. Total protein was isolated from shoots of pea grown for 120 h in TC-supplemented perlite (250 mg × L^−1^) or perlite with no TC (control plants). The 2D electrophoretic maps of proteins from non-TC shoots contained 326 spots, whereas maps of shoot proteins from TC-treated seedlings contained only 316 spots. The identity of 26 proteins was determined. The intensity of most proteins (62%) increased. This was particularly visible with diphosphate kinase, superoxide dismutase [Cu-Zn], peroxiredoxin, and glutathione S-transferase. A distinctly increased quantity of a protein involved in photosynthesis (photosystem II stability/assembly factor HCF136) was also noted. One protein was detected only in shoots of TC-treated plants (as opposed to controls); however, it could not be identified. Moreover, at the highest concentration of TC (250 mg × L^−1^ of perlite), a sharp increase in free-radical content was observed along with the amount of callose deposited in vascular bundles of leaves and roots and the occurrence of masses of dead cells in roots. It was found, therefore, that tetracycline which has been known for inhibiting predominantly the attachment of aminoacyl-tRNA to the ribosomal acceptor in bacteria can disturb diverse metabolic pathways in plants.

## 1. Introduction

Significant amounts of contaminants, including heavy metals, aromatic hydrocarbons, solvents, plant protection chemicals, and drugs (veterinary and human) are constantly dispersing from the technosphere into ecosystems and agrocenoses [1]. As reported by [2], it is estimated that approximately, 100,000 tonnes of antibiotics are produced worldwide. A report published by the European Medicines Agency [3] concluded that, in 2014, 8,935.5 tonnes of antibiotics were used in 29 EU member states, of which 581.3 tonnes were used in Poland.

In 2014, within the EU member states, the most frequently used antibacterial drug was tetracycline [3], which is favoured for the low production costs, low toxicity, and a broad-spectrum of affected bacteria [4, 5]. The ribosomal acceptor of aminoacyl-tRNA is believed to be the main target of tetracycline-related antibiotics in bacteria [6]. Tetracyclines are poorly absorbed in the gastrointestinal tract of animals, and large parts of them avoid metabolic modifications and are excreted in faeces (to 60%) and urine (approx. 30%), eventually reaching the fields [7]. The contents of chlortetracycline, oxytetracycline, and tetracycline in manure amount to 46 mg × kg^−1^, 29 mg × kg^−1^, and 23 mg × kg^−1^, respectively [8]. However, the contents of chlortetracycline, oxytetracycline, and tetracycline in swine slurry amount to as much as 139.4 mg × kg^−1^, 354.0 mg × kg^−1^, and 98.2 mg × kg^−1^, respectively [9].

The inefficiency of tetracycline removal from wastewater results in this drug spreading in the environment [10, 11, 12]. In addition, tetracycline has a long metabolic half-life, ranging from 55 to 105 days [13]. The uptake and toxicity of antibiotics manifested by the limitation of growth and development of various cultivated plant species are well documented [14, 15, 16].

Our previous studies [17] have also demonstrated the uptake of tetracycline by pea and its transport to the above-ground parts of plants and subsequent storage in leaves. Plants have developed numerous defence mechanisms which protect the cell against damage caused by external stress factors [18]. In the antioxidative enzymatic system, inter alia catalase and peroxidases have been identified [19]. A change in the activity of oxidative stress enzymes under the influence of certain antibiotics has been demonstrated inter alia for peas, lentils, and soybeans [20, 21]. It is worth noting that the majority of enzymes involved in the detoxification in plants are not only present in the form of several enzymes of a particular type but also in the form of numerous isoforms [22]. An important mechanism of ROS removal is the ascorbate-glutathione cycle or superoxide dismutases, which are the only plant enzymes able to decompose O_2_^˙−^ [23, 24].

The physiological role of certain proteins involved in plant resistance to drought, salt, or thermal stress is relatively well known [25, 26, 27, 28]. On the other hand, responses of plant proteomes to soil antibiotics have not been well documented. The aim of this study was to identify proteins which differ significantly in their response to soil contamination and to characterize the changes in intracellular quantities of such proteins.

## 2. Materials and Methods

### 2.1. Materials

Pea seeds (*Pisum sativum* L.) cv. Cysterski (Poznańska Hodowla Roślin, Tulce, Poland) were germinated in seed germination paper (PHPU Eurochem BGD, Poland). Seven-day-old seedlings were transferred to pots (3 seedlings in each pot) filled with 200 ml of perlite (size 2–6 mm; Agro Perlite, Poland). The cultivation was carried out for seven subsequent days under 12 hours of light, at temperatures of 23°C and 16°C, respectively, during the day and at night. The illumination intensity was 8 kLx. 14-day-old plants with a height of 10-11 cm were selected, and aqueous solutions of tetracycline (Sigma-Aldrich) were added to pots in concentrations of 0, 0.5, 5, 10, 50, and 250 mg × L^−1^ of the substrate. The control seedlings were watered with distilled water. On a one-off basis, 15 ml of either TC solution or water were added to the pot. The above-ground parts of the seedlings were sampled for analyses after 24 hours and after 120 hours (5 days) from the application of tetracycline. Plants growing for 120 h with TC were watered with 15 ml distilled water after 24 h, 48 h, and 72 h of growth with TC. After the experiments, the contaminated soil was taken by the Waste Treatment and Disposal Department of the Warmia and Mazury University in Olsztyn.

Green colour of the leaves (a minimum of 20 measurements for a particular variant) was determined using a Minolta SPAD-502 chlorophyll meter, while the osmotic pressure of the xylem sap in the plants was determined using a stationary measurement device, i.e., a Scholander pressure bomb (model 300).

### 2.2. Protein Isolation and Separation by the 2D Electrophoresis Method

For the analyses, seedlings which had grown for five days with tetracycline at a dose of 250 mg × L^−1^, as well as control seedlings (0 TC mg × L^−1^ of perlite), were used. Protein isolation was carried out following Badowiec et al. [29]. The above-ground parts of pea seedlings were ground to a powder in liquid nitrogen. Protein isolation and separation by the 2D electrophoresis method was carried out according to Dobiesz and Piotrowicz-Cieślak [30]. The peptide separation and determination of peptide masses was carried out using the Orbitrap spectrometer (Thermo). The measured peptide masses were compared to the records from the Viridiplantae database using MASCOT software.

### 2.3. Chlorophyll Absorption and Fluorescence Spectra Testing

The absorption and fluorescence spectra of chlorophyll isolated from pea leaves were measured 24 hours and 120 hours after the application of tetracycline. 300 mg of pea leaves were homogenised in a mortar with 5 ml of 96% ethanol. After centrifugation at 1700 g for 15 min, the supernatant was diluted 6 times, and then, the supernatant was subjected to analyses of absorption spectra using a Carry 300 UV-Visible Spectrophotometer (Varian, Inc.) and of the fluorescence spectra using a Carry Eclipse Fluorescence Spectrophotometer (Varian, Inc.). The excitation slit width was 5 nm, and the emission slit width was 2.5 nm for all measurements. With all samples, excitation was at *k*_exc_ = 650 nm. Absorption and emission spectra were determined at room temperature.

### 2.4. Guaiacol Peroxidase Activity

Guaiacol peroxidase activity [31] was determined in (200 mg) shoots homogenised on ice in a buffer for isolation with the following composition: 0.1 M Tris-HCl (Sigma-Aldrich), 8.75% polyvinylpyrrolidone (Sigma-Aldrich), 0.1 M KCl (PPH Stanlab), and 0.28% Triton X-100 (Sigma-Aldrich). The extract was centrifuged at 1700 g at a temperature of 4°C for 30 minutes. The amounts of isolated proteins were determined using the method in [32]. Guaiacol peroxidase activity was measured by the spectrophotometric method (CECLI, CE2021 2000 series) at the wavelength of *λ* = 470 nm, at room temperature. The reaction mixture contained 50 *μ*l of protein extract, 25 *μ*l 0.06% H_2_O_2_, 0.1 M KH_2_PO_4_ (pH 7, Chempur, Poland), and 100 *µ*l of 1% guaiacol (*o*-methoxyphenol; Sigma-Aldrich). One unit (U) corresponds to the oxidation of 1 *µ*M H_2_O_2_ during a period of 1 minute per 1 g of protein.

### 2.5. Microscopic Analyses

#### 2.5.1. Stereoscopic Microscope

Pictures of lateral roots and stipules of the pea five days after the application of the highest TC concentration (250 mg × L^−1^) and of control seedlings were taken using a Leica M205 C stereoscopic microscope at a magnification of 7.8x.

#### 2.5.2. Confocal Microscope

Control seedlings and the seedlings cultivated on the substrate with tetracycline at a concentration of 50 mg × L^−1^ were subjected to analyses in order to detect the presence of reactive oxygen species (ROS), dead cells, and callose 24 hours after the addition of the antibiotic. For the analyses, a confocal microscope (Leica TCP SP5) was used.

The 2′,7′-dichlorodihydrofluorescein diacetate (H_2_DCF-DA) was used as an indicator for ROS. The samples were placed in an H_2_DCF-DA in 0.1 M phosphate-buffered saline (PBS) in the dark. After 30 min, the medium was replaced with fresh PBS. Propidium iodide (PI) as an aqueous solution (10 *μ*g/ml) was used to identify nonviable cells within living plant tissue [33]. Seedlings were stained for 5–15 minutes in the dark and rinsed in water. For callose staining, samples were fixed in 99.8% ethanol, rehydrated (75%, 50% ethanol), placed in 150 mM K_2_HPO_4_ containing 0.05% aniline blue (AB) solution for 3-4 hours, and rinsed in 150 mM K_2_HPO_4_ (pH 9). After staining, samples were analysed by confocal laser-scanning microscopy (Leica TCS SP5). In the experiment, the following excitation and collection wavelengths were used: for H_2_DCF-DA, 488 nm excitation and 515–565 nm emission, for PI, 561 nm excitation and 610–650 nm emission, and for AB, 405 nm excitation and 480–550 nm emission. Dual staining images were collected during sequential scanning of each fluorochrome. Images show overlay projections (snapshots collected from Z-series images) produced from sequential scanning and LAS software (Leica Application Suite 2.0.2 build 2038).

### 2.6. Statistical Analyses

One-way analysis of variance (ANOVA) followed by Tukey'a comparison post hoc test (*p* ≤ 0.01) was applied to evaluate differences between controls and treatments using to Statistica 6.0. Standard deviation (SD) was determined for all measurements. The number of replicates was from 5 (in biochemical analyses) to 20 (in chlorophyll level and osmotic potential determinations).

## 3. Results and Discussion

The following were analysed: the general appearance of seedlings, the leaf colour intensity, turgor pressure, proteomic changes, the presence of free radicals, callose, and dead cells, chlorophyll absorption spectra and its fluorescence, and the activity of guaiacol peroxidase in the seedling shoot.

### 3.1. General Appearance of Seedlings

**Figure 1:**
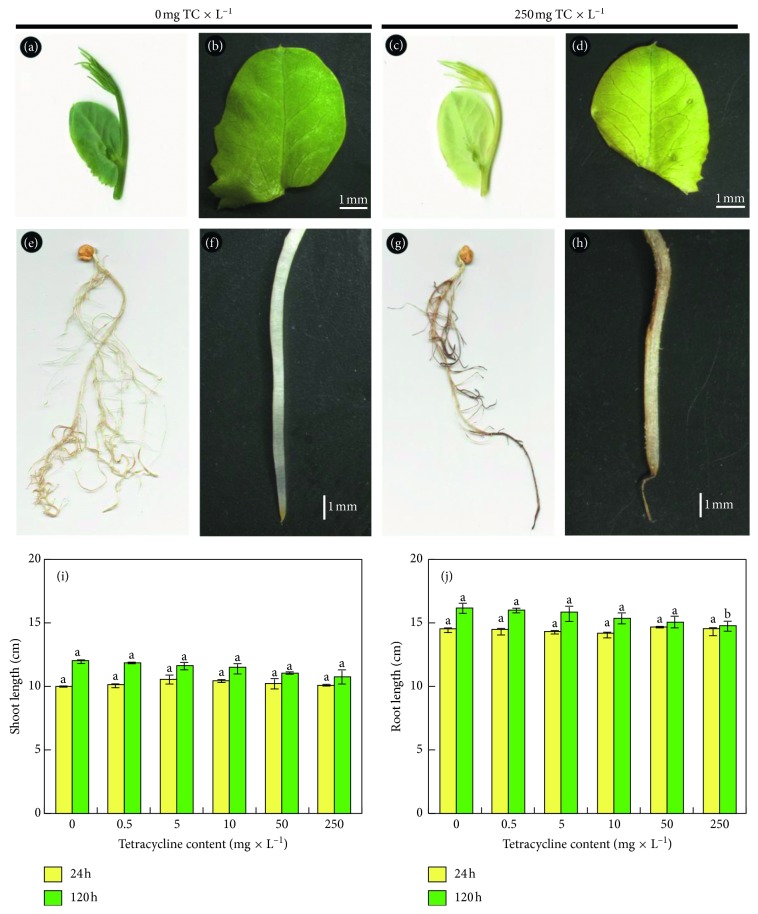
The general appearance of pea stipules from plants growing without tetracycline (a, b) or with 250 mg TC × L^−1^ of perlite TC (c, d). The roots of control seedlings (e, f) and TC‐affected seedlings (g, h). Root and shoot lengths (i, j) after 24 (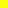) and 120 (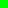) hours of growth in soil supplemented with different tetracycline concentrations (0: control, 0.5, 1, 5, 10, 50, and 250 mg × L^−1^ of perlite).

The pea plants growing for two weeks were from 10 to 11 cm tall. The height of seedlings did not change 24 hours after the application of TC to the substrate. Comparing pea seedlings growing for 120 h on a substrate containing 250 mg × L^−1^ TC with controls (seedlings watered with distilled water), differences in the length of the shoot and roots were noted, however not significant, amounting to 1 cm (Figure 1). In seedlings growing with tetracycline for 120 h, leaf chlorosis and necrosis of the apical part of the roots occurred. After 120 h of growth with TC, necrosis of the roots affected from 2.5 to 3 mm of the apical part of the main root and not more than 2 mm of the apical part of the lateral roots (Figure 1).

**Figure 2:**
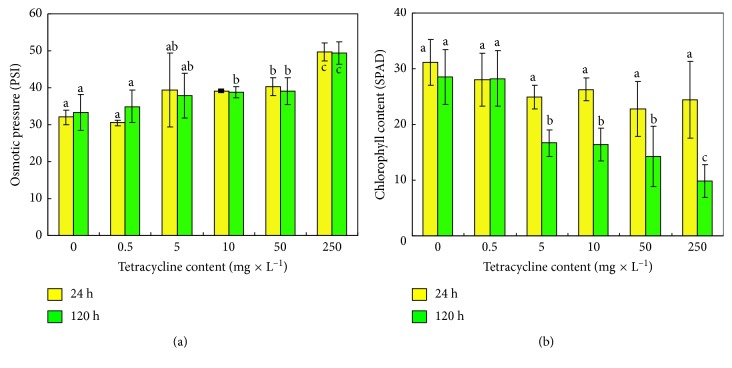
Osmotic pressure (PSI) (a) and green leaves index (SPAD) (b) of pea seedlings after 24 (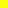) and 120 (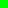 hours of growth in soil supplemented with different tetracycline concentrations (0: control, 0.5, 1, 5, 10, 50, and 250 mg × L^−1^ of perlite). Means with the same letter are not significantly different from each other (Tukey's test, *p* ≤ 0.01).

The presence of necrotic areas may result in a reduction in turgor pressure of the seedlings. A decrease in turgor pressure (i.e., xylem sap pressure) occurred as early as after 24 hours, regardless of the TC concentration applied. After 120 h, xylem sap pressure in the seedlings decreased by 5%, 14%, 16%, 17%, and 47%, respectively, for concentrations of 0.5; 5; 10; 50; 250 mg × L^−1^ of perlite compared to the control seedlings (Figure 2).

Plant exposure to antibiotics resulted in differences in colour intensity of the stipules. In order to measure these changes, a (Minolta) chlorophyll meter was used. The measurement of the stipule colour revealed 28 SPAD units for control leaves and 10 SPAD units for the leaves growing for 120 h with TC (Figure 2).

The uptake of antibiotics by plants has been repeatedly demonstrated by the authors of this paper [17] and other researchers [34, 35]; therefore, it was not analysed in this paper. The uptake of drugs from soil may be limited by the drug adsorption to the soil particles. In order to eliminate bonding with the soil, perlite was used as the substrate. One may reasonably speculate that the ionic activity in the growth substrate was minimal. The perlite substrate (size 2–6 mm; Agro Perlite, Poland), purely mineral, was used instead of a typical soil, rich in organic matter. Therefore, the soil biota of this substrate was not studied.

### 3.2. Analysis of the Proteome

**Figure 3:**
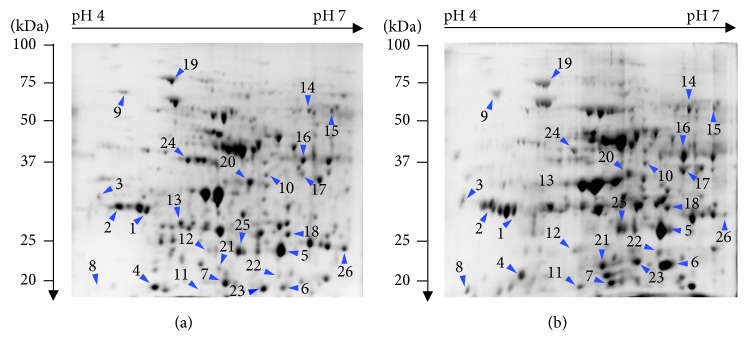
2D electrophoresis of shoot proteins from seedlings grown without (a) and with (b) tetracycline at the concentration of 250 mg × L^−1^ of perlite. Protein separation was conducted at pH 4‐7.

In order to obtain information on plant responses to stress and their molecular determinants, differences in the level of protein accumulation were measured in control shoots and shoots growing with tetracycline at the highest concentration. Only the above-ground plant parts were analysed as shoots as they have no direct contact with TC. A comparative study on shoot proteomes allows the determination of the consequences of tetracycline-induced stress. Proteins were isolated from the shoots of pea growing for 120 h without TC and with the highest TC dose. In the electrophoretic image without TC, 326 spots were obtained, i.e., 10 more than that obtained for the seedlings growing with TC (Figure 3).

**Figure 4:**
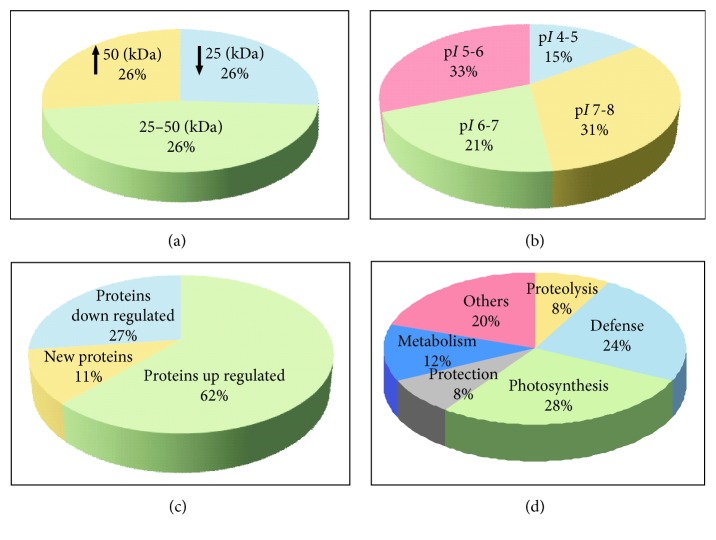
Pie chart showing the molecular mass distribution (kDa) of proteins from control shoots (a) and the tetracycline-affected shoots (b) at the concentration of 250 mg × L^−1^ of perlite and function of proteins from control roots (c) and the tetracycline-affected shoots (d) at the concentration of 250 mg × L^−1^ of perlite.

Most spots, i.e., almost 50%, had a molecular weight of 25 to 50 kDa and a p*I* value ranging from 5 to 6 (Figures 4(a) and 4(b)). As regards the proteins separated by the 2D electrophoresis method, 26 spots showed the greatest differences of the optical density between the control sample (without TC) and the plants with TC. The identified proteins are presented in Table 1. In terms of functionality, proteins involved among others in the processes of photosynthesis, protection, defense, and cellular respiration can be distinguished among the identified proteins (Figure 4(c)).

The percentages of spots whose intensity increased and decreased under the influence of tetracycline as compared with the control seedlings are presented in Figure 4(d). The intensity of 62% of the identified spots increased under the influence of tetracycline, particularly diphosphate kinase (a signal protein, whose accumulation under the influence of tetracycline increased the most). In addition, an increase in the intensity in samples exposed to tetracycline was noted for defensive proteins: (superoxide dismutase [Cu-Zn], peroxiredoxin (2 spots), and glutathione S-transferase; proteins involved in the degradation of damaged proteins (20S proteasome and ATP-dependent Clp protease); metabolic proteins involved in the biosynthesis of fatty acids (enoyl-acyl-carrier-protein reductase (NADH)); proteins involved in the process of photosynthesis (photosystem II stability/assembly factor HCF136); carbohydrate metabolism, i.e. fructose-bisphosphate aldolase (2 spots); and, ripening protein and ribonucleoprotein.

**Figure 5:**
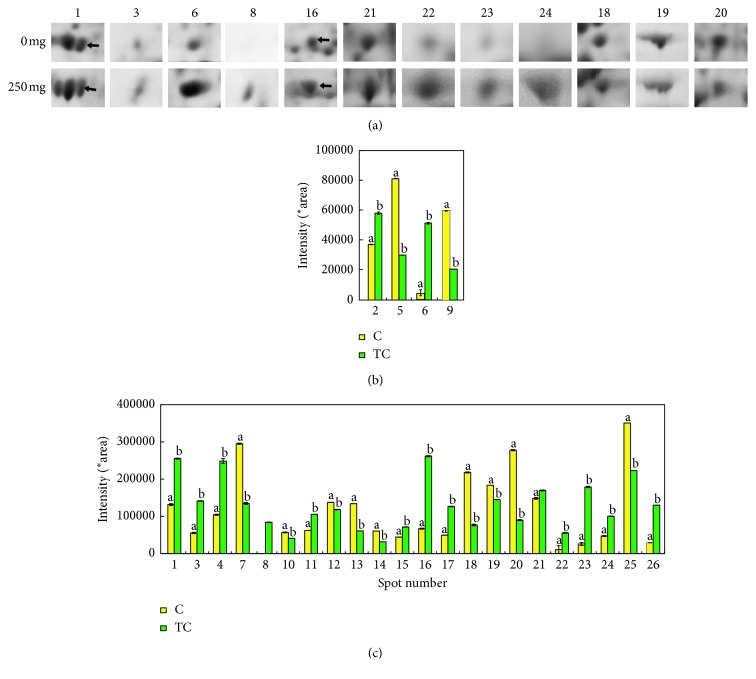
(a) Plain view of selected spots on 2D gels. (b, c) The average intensity of specific protein spots (PDQuest; BioRad) for control shoots (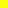) and tetracycline-treated shoots (250 mg × L^−1^ of perlite) (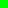); spot numbers: 1, 2, 3,… (compared with Table 1).

A comparison of the images of selected spots with diagrams of intensity is presented in Figure 5. The greatest differences were observed for diphosphate kinase (a 13-fold increase under the influence of tetracycline), superoxide dismutase [Cu-Zn] (a 7-fold increase), and peroxiredoxin (an almost 6-fold increase). On the other hand, a three-fold drop in accumulation was demonstrated for dehydrin and ascorbate peroxidase. Of the 26 proteins identified by the MALDI TOF/TOF MS method, certain identified proteins were present as isoforms: oxygen-evolving enhancer protein 2, chloroplastic (3 spots), ribonucleoprotein (2 spots) or ATP synthase CF1 (2 spots), peroxiredoxin (2 spots), and ABA-responsive protein (2 spots). This phenomenon is common for 2D electrophoresis, which results from the presence of various isoforms or degradation in the protein extracts; they can also be related to allelic polymorphism [36].

As regards the identified proteins, particular attention was paid to the proteins directly related to stress. A significant role in plant resistance to stress is attributed to diphosphate kinase. A study conducted by [37] demonstrated that the overexpression of diphosphate kinase gene is related to the increased expression of the genes of oxidative stress enzymes, e.g., peroxidase, glutathione transferase, thioredoxin reductase, peroxiredoxin, and protective genes encoding several heat-shock proteins. In the seedlings growing with TC, the expression of this protein was definitely higher than in control seedlings. An increase in the accumulation of superoxide dismutase [Cu-Zn], glutathione transferase, and peroxiredoxin under the influence of tetracycline was demonstrated. Peroxiredoxin has been recognised as an antioxidative enzyme associated with salt stress [38], and it is also probably involved in the resistance to stress induced by antibiotics in the substrate. An interesting fact is that our study demonstrated a decrease in the accumulation of ascorbate peroxidase under the influence of tetracycline. An increase in accumulation of enzymatic proteins responsible for antioxidative responses, including ascorbate peroxidase, has been previously noted in plants affected by heat stress [39], low temperatures [40, 41], or stress induced by the presence of sparfloxacin [21]. Other antibiotics may induce similar patterns of biochemical changes in plants, with additional disturbances in some cases, e.g., the action of sulfamethazine on plants results in impairments of mitochondria [20].

In pea shoots, there was a 1.5-fold decrease in the accumulation of HSP 70 under the influesnce of tetracycline. HSP 70 proteins are accumulated during salt or heat stress or during drought [26, 27, 28]. On the other hand, the content of HSP 70 in pea shoots decreased. A marked decrease in HSP 70 was observed during sparfloxacin and osmotic stress [21, 42]. Proteasome 26 protein complex (which contains subunit 20) plays a role in the control of proteolysis of damaged, misfolded, and oxidised proteins [43]. An increase in the accumulation of this protein and ATP-dependent Clp protease under the influence of tetracycline may indicate damage to the protein structures, induced by the antibiotic applied. An increase in the accumulation of ATP-dependent Clp protease degrading damaged proteins has so far been demonstrated in barley during a drought [44].

Under the influence of TC, the green colour of leaves disappeared, which undoubtedly affected the photosynthesis. The level of accumulation of proteins associated with the photosynthesis in the samples with tetracycline differed significantly from that of the control sample. Moreover, a protein was identified which was only found in the shoots subjected to the action of TC. An increase in the accumulation of both fructose biphosphate aldolase and certain proteins associated with the photosynthesis was observed in [45] for thermotolerant *Medicago sativa* L. under the influence of heat stress. In addition, the level of photosystem II stability/assembly factor HCF136 protein almost doubled.

#### 3.2.1. Chlorophyll Absorption and Fluorescence Spectra

**Figure 6:**
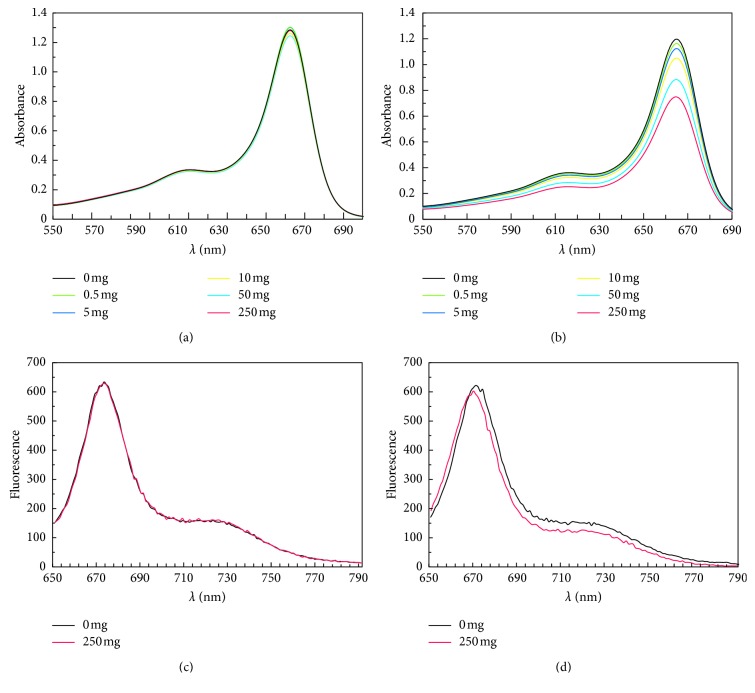
Absorption spectra of chlorophyll isolated from leaves of plants grown for 24 h (a) and 120 h (b) in perlite containing TC at the following concentrations: 0 (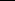), 0.5 (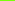), 5 (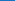), 10 (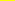), 50 (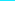), and 250 (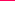) mg × L^−1^ of perlite. Fluorescence spectra of chlorophyll extracted 24 h (c) and 120 h (d) after tetracycline application: 0 mg × L^−1^ (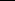) and 250 mg × L^−1^ of perlite (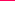).

The changes in leaf colour and in photosynthetic proteins stimulated the authors to analyse chlorophyll absorption and fluorescence spectra.  Figure 6 shows the absorption spectrum of chlorophyll a from the leaves of pea seedlings after 24 h (Figure 6(a)) and 120 h (Figure 6(b)) from the addition of tetracycline to the substrate. The tests were performed for all antibiotic concentrations applied. 24 hours after the addition of TC to the substrate, no differences in absorption spectra for any of the concentrations applied (compared with the control sample, which was significant after 120 h) were observed. The smallest differences (a decrease in the absorption by 0.05 in the absorption maximum at a wavelength *λ* = 665 nm) are visible for as low a concentration of perlite as 0.5 mg × L^−1^. With the increase in TC concentration, a decrease was observed in the absorption of chlorophyll a by 0.07, 0.13, 0.27, and 0.39 at a wavelength *λ* = 665 nm in relation to the control sample. The greatest decrease in the chlorophyll a absorption band (by 32% in relation to the control sample) was noted for the leaves growing with 250 TC mg × L^−1^ of perlite. The chlorophyll content of the leaves ranged from 1.16 × 10^−4^ to 0.73 × 10^−4^ M which corresponds to chlorophyll content ranging from 1.7 mg × g^−1^ fresh weight to 1.06 mg × g FW (Table 2). After 120 h of growth of the pea on the substrate containing 250 TC mg × L^−1^, the chlorophyll content of the leaves decreased by 37%; only 63% of chlorophyll a was left.

An analysis of fluorescence spectra was carried out for all tetracycline concentrations applied and for the control sample. Figures 6(c) and 6(d) present the fluorescence spectra for the chlorophyll of the leaves of the pea without TC and for 250 TC mg × L^−1^ of the substrate. 24 hours after the addition of TC to the substrate, no differences were observed in the position of the spectrum maximum for any of the concentrations applied as compared with the control. After 120 h, there was a shift of the maximum of the chlorophyll a fluorescence band by 2 nm towards shorter waves for 250 TC mg × L^−1^. The shift concerned the band at shorter wavelengths. In addition, 120 h after the addition of the antibiotic to the substrate, a decrease in fluorescence emission by 20 was recorded as compared with the control for the seedlings subjected to the action of tetracycline at the highest concentration.

The analysis of absorption spectra was limited to chlorophyll a, a band with a wavelength *λ* = 665 nm. The choice of this band was motivated by the fact that shorter waves incorporate the tetracycline spectrum which superimposes on the chlorophyll spectrum. Clearly visible changes in chlorophyll a absorption spectra after 120 h indicate a decrease in chlorophyll content under the influence of tetracycline. A decrease in chlorophyll concentration was observed under the influence of lead for *Phaseolus mungo* L., *Lens culinaris* L. [46], a case of salt stress for *Phaseolus vulgaris* L. [47], or a drought for *Matricaria chamomilla* L. [48].

Chlorophyll fluorescence has been used as a powerful, nondestructive, and reliable tool in plant physiology for understanding the primary events of photosynthesis and the effects of stress on photochemistry [49]. The shift of the maximum of fluorescence spectrum by 2 nm 120 h after the application of TC indicates the formation of a new product. Moreover, none of the studies conducted so far on the effects of stress demonstrated changes in the position of the fluorescence maximum.

The free radicals generated during the action of TC on the plant probably degrade chlorophyll and change the position of the fluorescence maximum. Salehi et al. [48] indicate that free radicals cause peroxidation and thus chlorophyll pigment degradation. This is why reactive oxygen species, callose, and dead cells were located in the roots.

#### 3.2.2. Location of Reactive Oxygen Species, Callose, and Dead Cells

Reactive oxygen species were detected using non-luorescent 2′,7′-dichlorodihydrofluorescein diacetate (H_2_DCFDA), freely penetrating into the cells. This compound is degraded inside the cells to a highly fluorescent 2′,7′-dichlorofluorescein (DCF), which bonds with ROS, and is detected using a microscope.

**Figure 7:**
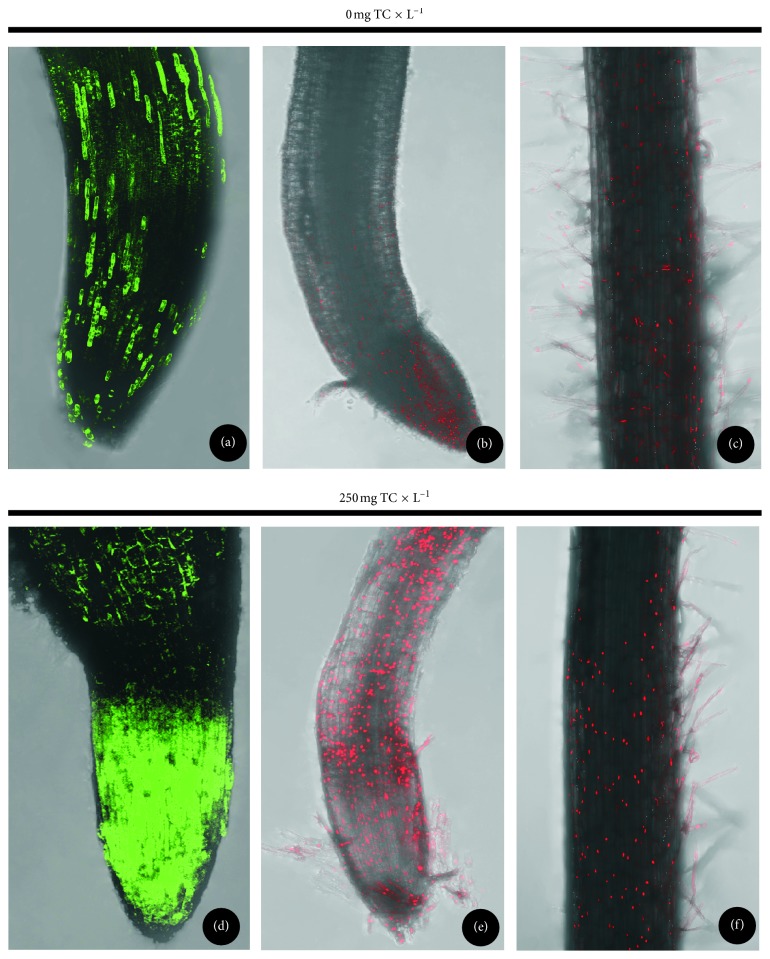
The distribution of ROS (a, d) and dead cells in root tips (b, e) and within the root hair zone (c, f) of control pea (a‐c) and pea subjected to TC treatment (at the concentration of 250 mg × L^−1^ of perlite; d‐f).

The photographs are a complete set of images from particular specimen layers, superimposed upon each other (Figures 7(a) and 7(d)). There were differences in the ROS content of the apical zone of the roots of seedlings exposed to the action of tetracycline for 24 hours, at a concentration of 30 mg × kg^−1^ of the soil weight as compared with the control sample. TC resulted in the generation of ROS. The intensity of fluorescence in the roots with tetracycline was higher; in addition, the narrowing of the apical zone of the roots was observed. In control seedlings, few cells containing free radicals (with a lower density as compared with the seedlings subjected to the action of the antibiotic) could be seen.

Dead cells were located in pea tissues using propidium iodide. Propidium iodide only penetrates through a damaged cell membrane, staining nucleic acids and causing luminescence in red. A smaller number of stained cell nuclei were observed in control seedlings as compared with the roots of seedlings exposed to tetracycline (Figures 7(c)–7(f)). In addition, dead cells were located both in the apical zone and the root hair zone of the roots and in the root hairs themselves. In the apical zone of the roots subjected to tetracycline, delamination of the rhizodermis cells can be seen.

**Figure 8:**
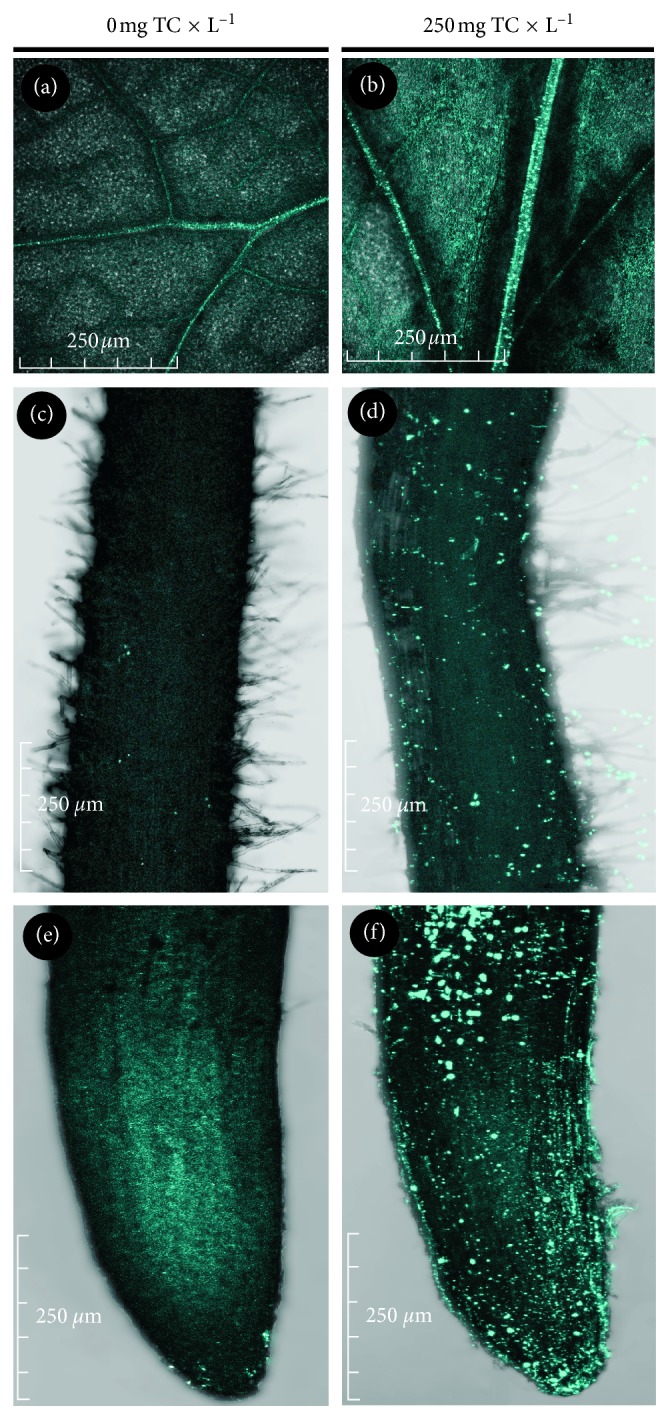
Callose location in leaves (a, b) and within the root hair zone (c, d) and in root tips (e, f) of control pea (a‐c) and pea subjected to TC treatment (at the concentration of 250 mg × L^−1^ of perlite; d‐f).

Layers of callose were tested by staining the roots and leaves with aniline blue. The fluorescence indicating the presence of callose layers in the tissues was located in the apical zone of the roots and in the root hair zone (Figure 8). The obtained results indicate a higher callose content of the tissues subjected to the action of TC. In the leaves, intense luminescence is primarily seen in the vascular bundles of the leaves and, to a lesser extent, in the parenchyma.

The generation of ROS in plants is triggered by different kinds of environmental stresses, such as high light, high or low temperature, salinity, drought, nutrient deficiency, and pathogen attack [50]. Pollution of agricultural soils with pharmaceuticals and personal care products results in overproduction of ROS in plants and promotes the impairments of cell membranes and lipid peroxidation, pointing to the occurrence of oxidative stress. Increased ROS production was also observed in *Arabidopsis thaliana* subjected to bisphenol A which caused a disorganization of the thylakoid system and stroma [51, 52]. Strict control of the ROS level is necessary in order to prevent their toxic action on the cells and to ensure the proper performance of signal functions [22]. To ensure survival, plants have developed efficient antioxidant machinery [53]. The removal of excess free radicals is the main method of detoxification. Noctor and C. H. Foyer [54] recognised guaiacol peroxidase as one of the most important antioxidative enzymes in plant cells.

#### 3.2.3. Guaiacol Peroxidase Activity

Measuring the increase in absorbance, the activity of oxidative stress POX enzyme was tested in the above-ground parts of the pea plant as well as the roots of pea seedlings subjected to the action of TC. Tests were conducted both 24 hours and five days after the application of the antibiotic.

**Figure 9:**
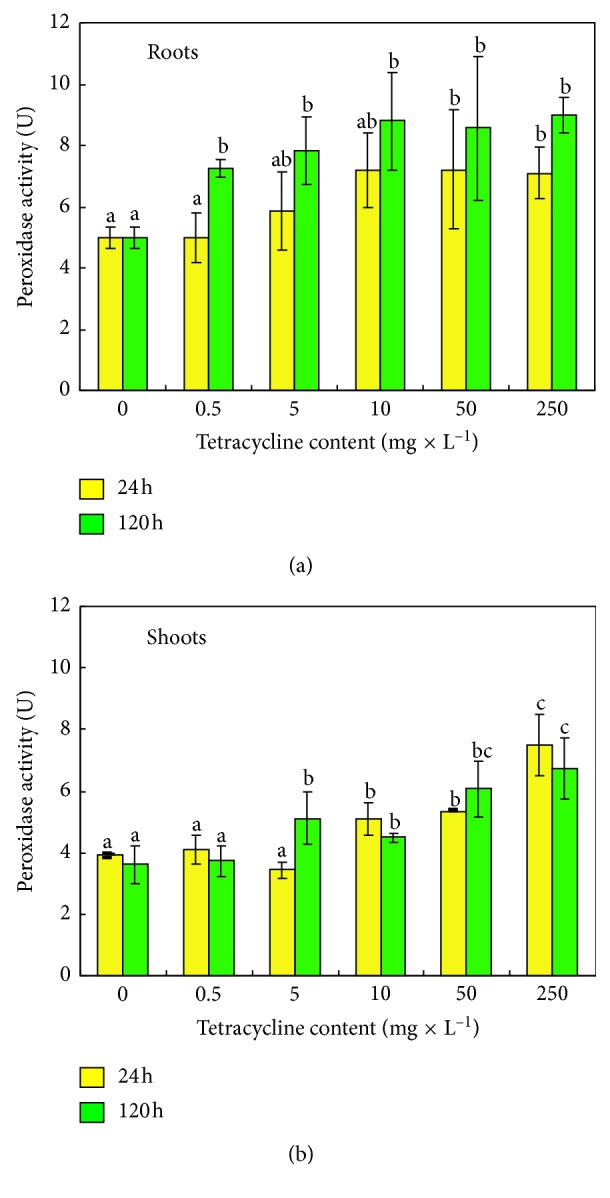
Activity of guaiacol peroxidase (U, one unit of enzyme activity corresponds to the oxidation of 1 mM H_2_O_2_ for 1 minute) in roots (a) and in shoots (b) of pea 24 h (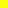) and 120 h (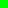) after the application of tetracycline. Means with the same letter are not significantly different from each other (Tukey's test, *p* ≤ 0.01).

The tests demonstrated an increase in guaiacol peroxidase activity in relation to the control seedlings both 24 and 120 hours after the application of TC (Figure 9). An increase in the activity of the enzyme was observed in the above-ground parts of the seedlings and in the roots. The lowest increase in POX activity (by 3% in relation to the control) in the seedling shoots was noted 24 hours after the addition of tetracycline to the substrate under the influence of the lowest of the antibiotic concentrations applied. An almost two-fold increase in POX activity was noted in the above-ground part of the pea plant under the influence of tetracycline at a concentration of 250 mg × L^−1^ of perlite. For the roots, 24 hours after the application of tetracycline, no change in POX activity was observed in a dose of 0.5 mg × L^−1^ of perlite. With an increase in the antibiotic dose applied, the activity level increased. For subsequent concentrations, an increase, in the following order, by 17%; 44%; 44%; and 42% was noted in relation to 5, 10, 50, and 250 mg × L^−1^ perlite, respectively. The data confirm that antibiotic soil pollutants stimulate the activity of POX in plant tissues. POX is known as one of the main components of secretome of plants challenged by environmental stresses [55, 56]. Peroxidases play many important roles in plant metabolism. They are active in the regulation of plant development and growth as well as in detoxication processes and stress responses [57]. Guaiacol peroxidase, the enzyme related to important biosyntheses and defence against biotic and abiotic environmental challenges, is considered a “stress-related enzyme” [55]. POD appears in plant tissues whenever ROS accumulation occurs. POD scavenges ROS; on the other hand, it stimulates their production [58]. POD is also a key player in the process of deactivation of toxins [59]. Agostini et al. [60] have shown that peroxidases are able to degrade 2,4-dicholorophenol (a pesticide). It was also demonstrated that peroxidases can oxidize diclofenac [61]. The contamination of agricultural soils with pharmaceutical and personal care products leads to the increase in POD activity in roots and leaves, corresponding to the dose and exposure time [52]. Both the abovementioned studies and our own results clearly show that POD may play a significant role in drug transformation in plants.

## 4. Conclusions

Treatment of pea seedlings with tetracycline (applied to the growth substrate at up to 250 mg × L^−1^) did not result in any deteriorative changes visible within 24 hours. After five days (120 h) of TC action, pea leaves lost 38% of their chlorophyll which resulted in their discoloration. Moreover, the proteome of antibiotic-affected plants contained 10 proteins less than the proteome of control plants. On the other hand, the quantity of some proteins increased as a result of tetracycline action. These included chiefly diphosphate kinase, superoxide dismutase [Cu-Zn], peroxiredoxin, glutathione S-transferase, and a protein involved in photosynthesis (photosystem II stability/assembly factor HCF136). Moreover, at the highest concentration of TC (250 mg × L^−1^ of perlite), a sharp increase in free-radical content, the amount of the deposited callose and the occurrence of masses of dead cells were observed. It was found, therefore, that environmental tetracycline can disturb plant proteome, oxidative stress responses, and photosynthetic apparatus.

## Figures and Tables

**Table 1:** Proteins in pea shoots identified by LC-MS-MS/MS analyses.

Spot no.	Protein name	Species	Accession no.	Score	Protein sequence coverage (%)	Theoretical p*I*
1	20S proteasome subunit alpha	*Medicago truncatula*	XP_013470289.1	589	34	4.69
2	Ribonucleoprotein	*Pisum sativum*	CAA74889.1	1149	45	4.82
3	ATP-dependent Clp protease proteolytic subunit 4, chloroplastic	*Arachis ipaensis*	XP_016201235.1	273	16	5.41
4	Peroxiredoxin-2E, chloroplastic-like	*Lupinus angustifolius*	XP_019426240.1	302	12	7.77
5	Oxygen-evolving enhancer protein 2, chloroplastic; Short = OEE2; AltName: Full = 23 kDa subunit of oxygen evolving system of photosystem II; AltName: Full = 23 kDa thylakoid membrane protein	*Pisum sativum*	P16059.1	1514	59	8.29
6	Diphosphate kinase 2, chloroplastic	*Pisum sativum*	P47923.1	510	31	8.49
7	ABA-responsive protein ABR17	*Pisum sativum*	Q06931.1	608	53	5.07
8	Uncharacterized protein	*Solanum lycopersicum*	XP_004253362.1	202	25	5.06
9	Ribonucleoprotein	*Pisum sativum*	CAA74889.1	484	24	4.82
10	Enoyl-acyl-carrier-protein reductase (NADH), chloroplastic-like	*Cicer arietinum*	XP_004505719.1	1074	48	8.98
11	ABA-responsive protein ABR17	*Pisum sativum*	Q06931.1	848	70	5.07
12	Uncharacterized protein	*Glycine max*	NP_001235072.1	588	33	5.20
13	Glutathione S-transferase	*Pisum sativum*	BAC81649.1	580	36	
14	ATP synthase CF1 alpha subunit	*Pisum sativum*	AIK21447.1	1695	42	5.92
15	ATP synthase CF1 alpha subunit	*Pisum sativum*	AIK21447.1	1617	47	5.92
16	Fructose-bisphosphate aldolase 2, chloroplastic	*Pisum sativum*	Q01517.2	1760	61	5.48
17	Fructose-bisphosphate aldolase 2, chloroplastic	*Pisum sativum*	Q01517.2	1338	31	5.48
18	Ascorbate peroxidase, cytosolic	*Pisum sativum*	P48534.2	777	45	5.52
19	70 kDa heat shock-related protein, chloroplastic	*Pisum sativum*	Q02028.1	3644	58	5.22
20	Dehydrin-cognate	*Pisum sativum*	CAA78515.1	929	38	5.40
21	Ripening-related protein	*Pisum sativum*	AAQ72568.1	531	32	5.11
22	Peroxiredoxin	*Pisum sativum*	CAQ56034.1	373	24	5.57
23	Superoxide dismutase [Cu-Zn], chloroplastic	*Pisum sativum*	P11964.1	678	49	5.94
24	Low quality protein: photosystem II stability/assembly factor HCF136, chloroplastic	*Cicer arietinum*	XP_012572716.1	997	35	6.86
25	Oxygen-evolving enhancer protein 2, chloroplastic; Short=OEE2; AltName: Full = 23 kDa subunit of oxygen evolving system of photosystem II; AltName: Full = 23 kDa thylakoid membrane protein	*Pisum sativum*	P16059.1	1286	56	8.21
26	Oxygen-evolving enhancer protein 2, chloroplastic; Short = OEE2; AltName: Full = 23 kDa subunit of oxygen evolving system of photosystem II; AltName: Full = 23 kDa thylakoid membrane protein	*Pisum sativum*	P16059.1	769	36	8.21

**Table 2:** Chlorophyll concentration (*C*(M) ± standard deviation (SD), chlorophyll content in leaves (mg × g^−1^ ± SD), and the percentage fraction of chlorophyll concentration (*C*(%) ± SD) of plants grown in soil containing different amounts of TC (0.5 mg to 250 mg × L^−1^ of perlite).

Tetracycline content	24 hours after tetracycline application			120 hours after tetracycline application		
(mg × L^−1^)	*C*(M) ± SD	mg × g^−1^ ± SD	*C*(%) ± SD	*C*(M) ± SD	mg × g^−1^ ± SD	*C*(%) ± SD
0	1.16 × 10^−4^ ± 0.9 × 10^−5^	1.7 ± 0.13	100 ± 7.7	1.16 × 10^−4^ ± 0.9 × 10^−5^	1.70 ± 0.13	100 ± 7.7
0.5	1.16 × 10^−4^ ± 0.4 × 10^−5^	1.7 ± 0.05	100 ± 3.4	1.05 × 10^−4^ ± 0.2 × 10^−5^	1.54 ± 0.02	91 ± 1.2
5	1.16 × 10^−4^ ± 0.2 × 10^−5^	1.7 ± 0.02	100 ± 4.7	1.03 × 10^−4^ ± 0.1 × 10^−5^	1.50 ± 0.01	89 ± 0.6
10	1.16 × 10^−4^ ± 0.8 × 10^−5^	1.7 ± 0.11	100 ± 6.8	0.97 × 10^−4^ ± 0.7 × 10^−5^	1.42 ± 0.10	83 ± 5.9
50	1.16 × 10^−4^ ± 0.6 × 10^−5^	1.7 ± 0.08	100 ± 5.1	0.84 × 10^−4^ ± 0.4 × 10^−5^	1.23 ± 0.05	72 ± 2.9
250	1.16 × 10^−4^ ± 0.7 × 10^−5^	1.7 ± 0.10	100 ± 6.0	0.73 × 10^−4^ ± 0.1 × 10^−5^	1.06 ± 0.01	63 ± 0.6

## Data Availability

The data used to support the findings of this study are available from the corresponding author upon request.
